# Reviews, Notices, &c.

**Published:** 1857-09

**Authors:** 


### REVIEWS, NOTICES, &c.

THE *Evening Post* says, that "J. H. Higginson, 71 Maiden Lane, has just published a highly-finished NEW RAILROAD MAP, which he claims with justice to be the largest and best ever prepared in this country. Its greatest merit, probably, is its accuracy; every route having been compiled from maps furnished by railroad companies—a feature never before attempted in works of its class, and one which will be duly appreciated by travellers. There is not a route finished, commenced, or projected, between Maine and Texas, which is not here given, and as the information is the latest, so is the map the best and most intelligible to consult. The engraving and coloring are excellent."

We fully accord with the above, and add, that the map is beautifully colored, is about six and a half feet long, on rollers, with linen underground, making it as durable as it is convenient for reference. It will be sent, free of cost, to any part of the United States, for eight dollars.

It is also put up in Atlas form, twelve by fifteen inches, in sections, so as to be portable in a common travelling trunk, and is furnished at the same price. To persons who contemplate moving to the West, or desire to make investments there, this valuable map imparts information at a single glance, of the very highest importance, while to every intelligent traveller, the map in portable form, as a means of reference, reliable and satisfactory, is indispensable.

Mr. H. is preparing a map of the United States, intended to be the largest and best ever published. But it is of higher interest to New Yorkers that a map of the City of New York is in progress, to be completed within a year, which will be of great value to every property holder on the island, as it will give, in addition to ordinary maps, the original farm lines, showing at a glance, to each lot owner, the first proprietors' names, thus affording a key to title highly satisfactory to every purchaser. It will be after the manner of the *Brooklyn Farm Line Map*, prepared by Mr. H., and which the authorities have adopted.

BROWN'S GRAMMAR OF ENGLISH GRAMMAR. "*Noah Webster* has heretofore been claimed as indisputably the greatest of American authors; but we confess to a large degree of sympathy with those who claim for the author of the *Grammar of English Grammar*, the high distinction of being 'the greatest author of the nineteenth century.'"—*Illinois Teacher*. "We know of no work on this subject which equals this in merit."—*Advertiser*. Published by S. S. & W. Wood, 389 Broadway, New York.

LITTELL'S LIVING AGE. Boston, weekly, 8vo., $6 a year. Single numbers twelve cents. Edited with uniform ability, interest, and sound judgment.

BOSTON MEDICAL AND SURGICAL JOURNAL, weekly, $2 a year, is the favorite medical publication of New England, and the oldest.

RANKING'S ABSTRACT of Medical Progress, for the past six months, No. 25, vol. 13, published by Lindsay & Blakiston, Philadelphia, semi-annually, pp. 384, at $2.00, is not inferior to any of its predecessors, containing no less than 215 different articles.

Braithwaite's Half-Yearly Retrospect has been published seventeen years, in thirty-four parts, bound in 15 vols., sheep, library style, $2 a volume. The whole set, *delivered free of charge*, for thirty dollars. Stringer & Townsend, of New York, with characteristic enterprise, have published a complete alphabetical index of all the contents, from Part 1 to 34 inclusive, embracing the whole term of republication. With this *general index*, Braithwaite's Half-Yearly Retrospect forms for the young graduate, as well as for the old practitioner, the most desirable, complete and available medical library, for constant, convenient, and reliable reference, ever published. It gives a complete view of medical progress up to July of the present year, and to medical men is invaluable.

The Pacific, a Weekly Journal, devoted to Religion, Education, and Useful Intelligence, is edited with an ability and consistency, which, with its mechanical comeliness, and taking into account the local history and character of San Francisco, which, within a decade, was almost unknown to the civilized world—all these things make "The Pacific" the most remarkable "fact" of modern times. We trust it meets the success which it so largely merits.

The British and Foreign Medico-Chirurgical Review, or Quarterly Journal of Medicine and Surgery, republished at three dollars a year, *free of postage*, if paid in advance, continues to be "one of the cheapest, as well as best, of medical journals."

How to Write—How to Talk—How to Behave—How to do Business.—4 vols., sent by Fowler & Wells, 308 Broadway, New York, for one dollar; 30 cts. single. They are truly useful books—true, practical, and of permanent value.

The Impending Crisis of the South: How to Meet it. By Hinton Rowan Helper, of North Carolina. "Fourth thousand." 420 pp., 8vo. Published in good style by Burdick Brothers, 8 Spruce street, New York. This book is permanently valuable for the large amount of reliable statistical matter which it contains, which has been collected at great expense by its industrious and indiscreet author. The South will add to its prosperity by studying the statistics of this volume, for by so doing a spirit of inquiry will be aroused, which must lead to good results. We believe that the publishers, who are excellent men, will do the country a service by abridging the book, and leaving out all that Mr. Helper says himself, retaining only the statistics. He has failed to write with the courtesy which belongs to a Southern gentleman, irritating all, convincing none.

Putnam's Magazine. Lake George, Mendelssohn and his Music, Witching Times, Pistol Philosophy, Notes, Gossip, Humor, Little Joker, Glimpse at my Hotel, are amusingly instructive.

Blackwood's Magazine, for August, is of unusual interest.

